# Malignant Transformation in Diabetic Foot Ulcers—Case Reports and Review of the Literature

**DOI:** 10.3390/geriatrics4040062

**Published:** 2019-11-07

**Authors:** Stefan Dörr, Lara Lucke-Paulig, Christian Vollmer, Ralf Lobmann

**Affiliations:** 1 Department of Endocrinology, Diabetology and Geriatrics, Stuttgart General Hospital, 70374 Bad Cannstatt, 24 Prießnitzweg, Germany; l.lucke-paulig@klinikum-stuttgart.de; 2 Department of Orthopedics and Trauma Surgery, Stuttgart General Hospital, 70374 Bad Cannstatt, 24 Prießnitzweg, Germany; c.vollmer@klinikum-stuttgart.de

**Keywords:** Marjolin’s ulcer, chronic wound, wound healing, skin transplantation, matrix-metalloproteinases, Ackerman carcinoma, squamous cell carcinoma, SCC

## Abstract

An imbalance of regeneration and destruction of the extracellular matrix due to a plethora of chemo- and cytokines, elevated matrix metalloproteinases, bacterial contamination and repetitive painless tissue damage can lead the chronicity of a wound, especially in diabetic foot ulcers (DFU). Along general lines, wound healing and cancer development are similar. Therefore chronic wounds prepare a breeding ground for cancer development. Several characteristics such as increase in size, verrucous everted margins and contact bleeding are suspicious for malignant growth in a chronic wound. While previously the term Marjolin’s ulcer was attributed to a malignant tumor in (burn) scars, it is nowadays used for every malignant tumor in chronic wounds. Furthermore, chronic ulcers in diabetic feet are susceptible for malignant transformation. We describe two cases of squamous cell carcinoma in patients with DFU—a 71 year-old woman and a 67 year old man. Both received total tumor excision and split-skin grafts with good short-time results.

## 1. Introduction

### 1.1. Pathophysiology in Chronic Wounds

Leg and feet ulcers are a major health care burden and are an increasing problem in elderly during the last centuries due to a senescent population. The most common causes of chronic leg and feet ulcers are chronic venous insufficiency (CVI), peripheral arterial disease (PAD) and ulcers due to neuropathy in particular in individuals with long-standing diabetes. Diabetic foot ulcers (DFU) typically arise in areas with pressure load and are often caused by a mixture of PAD and neuropathy. 

**Figure 1:**
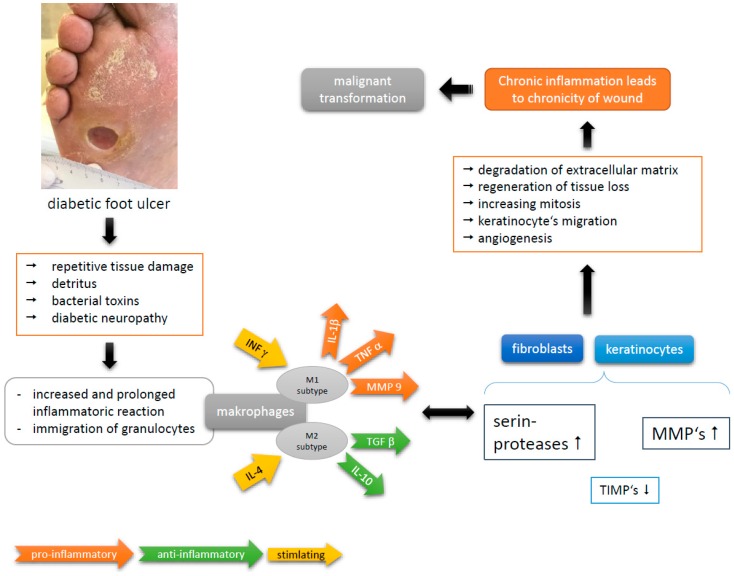
Pathophysiology of chronic wound in diabetic foot ulcers (DFU) (modified and based on [4,5]).

In chronic wounds, wound healing is disturbed because of a disproportion of regeneration and degeneration of extracellular matrix. Especially in diabetics, there is an intrinsic dysfunction of granulocytes with a deranged release of growth factors [1,2,3], for example, the level of platelet derived growth factor (PDGF) is low while that of matrix-metalloproteinases (MMP) is elevated. Especially, increased MMP-9 production is associated with poor wound healing [1]. This faulty microenvironment perpetuates a persistent degradation of extracellular matrix proteins [4,5,6]. Tumor necrosis factors (TNF) α and β, in addition to PDGF, raise mitosis rate and stimulate extracellular matrix production. To accomplish complete wound healing, an increase of inhibitors of MMP (TIMP-1, TIMP-2) is necessary [4]. However, persistently high levels of MMP lead to chronicity of a wound. This prolonged inflammatory reaction, especially in DFU, is further caused by bacterial contamination and repetitive painless tissue damaging [4]. Immigrated granulocytes stimulated by bacterial endotoxins, fragments of extracellular matrix and cellular detritus secrete a mixture of cytokines and growth factors. In particular, TNFα and interleukin 1β (IL 1β) lead to sustained chronic inflammation [5,7]. In this (micro)environment of chronic inflammation, stimulated mitosis, degradation and regeneration of extracellular matrix lies the cornerstone of abnormal cell proliferation. Therefore, inflammation is a key driver in development of cancer in chronic wounds (Figure 1) [5,8,9]. This is emphasized by several studies reporting small but significant lower risk of SCC in people taking nonsteroidal anti-inflammatory drugs, e.g., aspirin or ibuprofen [10].

The physiology underlying wound healing and cancer progression is quite similar along general lines, especially in epithelial cancers. The most critical phase in wound healing is the re-epithelialization, when keratinocytes initiate hyperproliferation and migration on the wound bed. Alterations in keratinocytes during re-epithelialization are rather similar to those that occur during cancer initiation and metastasis, with the crucial difference being that the hyperproliferative behavior is (normally) self-limiting in case of wound repair. For example, epidermal growth factor family (EGF) regulates cell proliferation and migration. There are further cytokines involved in wound healing and metastasis, such as fibroblast growth factor (FGF), transforming growth factor-β (TGF-β) and hepatocyte growth factor (HGF). Uncontrolled expression of MMPs facilitates metastasis and impairs wound healing as well [5].

The pathophysiology of neoplasms growing in chronic wounds has been discussed for about 100 years. Among the imbalance of cytokines in the microenvironment of chronic wounds, potential direct mutagenic effects of toxins released by necrotic tissue are discussed [11]. In addition, loss of immunologically active cells (like dendritic cells) in areas of chronic scar tissue are proposed. Hence, malignant cells evade immunological detection and become more aggressive and prone to metastasis [12]. Since no single specific factor has been identified yet, it is likely that pathogenesis is linked with multiple factors of environmental, immunological and genetic nature [13].

### 1.2. History of Marjolin’s Ulcer

The first author who described malignant lesions in burn scars was Aurelius Cornelius Celsus in the first century [14]. In 1828, the French physician Jean Nicolas Marjolin wrote about chronic ulcers arising in scar tissue in his “*Dictionnaire de Médicine*” [15]. He formed the term ‘Marjolin’s ulcer’, but he did not identify the relationship to malignancy. This relationship must be ascribed to the English surgeon Caesar Hawkins, who described skin cancer developing in burn scars and lacerations in his article “*On Warty Tumours of Cicatrices*” in 1833 [16]. Furthermore, Guillaume Dupuytren did not hesitate to use the term malignant ulcer when he reported the case of a 62 year old woman who had fallen on embers (burning coal) at the age of nine months and developed a fungoid mass on her cicatrized forearm later in her life [17]. In 1850, as an honor to Marjolin in the year of his death, the surgeon Robert William Smith (Trinity College, Dublin) first described ulcerations in burn wounds as a ‘warty ulcer of Marjolin’ [18]. Nowadays, all neoplasms growing in chronic wounds are embedded under the term Marjolin’s ulcer [19] regardless of their genesis.

### 1.3. Attributes of Marjolin’s Ulcer

Marjolin’s ulcer (MU) is a seldom, but often aggressive skin cancer that develops in skin areas with previous damage or chronic inflammation [13]. The incidence of MU in burn scares is denoted by 1%–2%. However, MU may also occur in other scar tissues (e.g., post-traumatic wounds) and in chronic wounds due to chronic osteomyelitis, neuropathy, venous insufficiency or pressure injury. Men are three times more frequently affected than women, with a preference of the fifth decade of life [13]. The risk of malignant transformation is highest in scars resulting from skin burn with 76.5% and decreases dramatically in chronic traumatic wounds (8.1%), venous leg ulcers (6.3%) and fistulas in context of chronic osteomyelitis (2.6%) [20,21,22]. About 60% of MU are found in the lower extremities and women are here affected twice as often as men [23]. Although in case of pressure ulcers the risk of malignant transformation is practically neglectable (0.5%) [24]. Some authors argue that MU of this origin have their own nature with more aggressive clinical features [25].

Based on the criterion of time MU could be distinguished in acute and chronic forms. The occurrence within 12 months from injury is referred to the acute form. The reported variation for malignant transformation ranges from 4 weeks up to 75 years [14]. Patients with depressed immune system have a greater susceptibility to malignant transformation [26].

**Figure 2:**
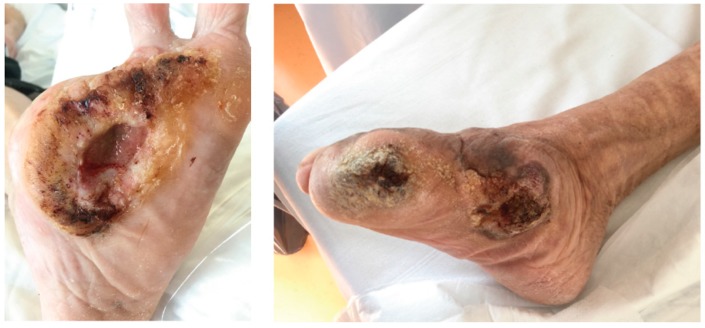
General findings in squamous cell carcinoma in DFU.

Indicative clinical findings for malignant transformation in a chronic wound include (Figure 2) [19]:

- nodule or verrucous formation;
- induration;
- everted margins;
- excessive granulation tissue;
- increase in extent during time;
- contact bleeding;
- absent tendency of healing within 3 months or more.

Pain is not a reliable marker in DFU because of a nearly always existing painlessness due to diabetic polyneuropathy. Therefore, to confirm diagnosis biopsy is obligatory.

The most frequent carcinoma found in burn scars and chronic wounds is a squamous cell carcinoma (**SCC**). Koval-Vern et al., for example, found SCC in 71% of 412 cases of skin burn neoplasms between 1923 and 2004 in his review of the literature [23]. Basal cell carcinoma (12%), melanoma (6%), sarcoma (5%) and other neoplasm (4%, e.g., malignant schwannoma) have also been identified [23]. Thus, the majority (71%) of Marjolin’s ulcers consisting of SCC and accounts for an incident of 1.5%–2.0% for the lower extremities [27,28].

The risk of neoplastic growth in vascular leg ulcers is rather low. Poccia et al. reported that about 2.4% of venous ulcers might develop malignant degeneration [29]. Among 155 examined leg ulcers in 145 patients, Senet et al. identified MU in 10.4% (nine cases of SCC, five cases of BCC) [30]. Risk factors for developing SCC in venous ulcers are advanced varicose veins, venous thromboembolism, chronic skin damage or ulceration and chronic infections. Exposure to sunrays rises des prevalence in favor of BCC [31].

### 1.4. Squamous Cell Carcinoma in Chronic Ulcers

Squamous cell carcinoma (SCC) at large is the second most frequent skin cancer and the most common carcinoma in scars and chronic wounds. SCC is defined as a malignant tumor of keratinocytes located in the epidermis and is characterized by infiltrative growth and frequent spreading into local lymph nodes. Occurring in chronic wounds or scars, SCC is attributed to be more aggressive than SCC of other origin or aetiology. The risk of metastases and recurrence is increased if not treated. At the beginning, it often appears as a small scaly bump or plaque progressing to a hard, protruded callus-like lesion [13,32,33]. A diffuse process at time of diagnosis is found in 32% of patients with malignant ulceration [13].

After confirming diagnosis of SCC, metastases must be excluded, because SCC of the lower extremities has a rate of metastasis of about 27%–30% [14,27,34]. Most metastases rise from primary tumors classified as Grade 2 or 3. SCC with high-risk characteristics include tumor size >2 cm, blurred margins, rapid growth, ulceration, poor differentiation, deep extension into subcutaneous fat and periwound or perivascular infiltration [35]. Recurrent SCCs have a greater risk of metastases. Distant metastases are mostly found in brain, liver, lung, kidney and distant lymph nodes [26]. Thus, metastases are the most important prognostic factor—affected regional lymph nodes reduce prognosis and lead to death within 2–3 years [13].

Staging and grading of SCC (Table 1) is composed of size, depth of infiltration through the histological skin layers, lymph node status and existence of metastases. The grading considers the percentage of differentiated cells in a sample of the tumor: G1 consists >75% of differentiated cells, G2 25%–75% and G3 < 25% of differentiated cells in the sample.

### 1.5. Diagnostic in MU

Whenever an MU is suspected, tissue specimens should be taken to confirm or exclude diagnosis. To increase the rate of accurate cancer diagnosis, standardized biopsy procedures should be established. Tissue specimens should be taken from various places of the ulcer and its margin and the examiner should be experienced in assess skin specimens. Thereby, it is possible to minimize false negative results [13].

After confirming diagnosis, the local and distant extent of the disease must be estimated by conducting x-rays of affected extremity and ultrasound examination of the anatomical lymphatic drainage region. Signs of osteomyelitis with diffuse demineralization and destruction on x-rays are usually consequence of bone involvement. It might be much more difficult for pressure ulcers in sacral or iliac areas to estimate bone involvement. Computer tomography (CT) is able to give more information in these areas. Compared with CT, magnetic resonance imaging (MRI) is considered as the best method in SCC to assess the level and extent of bone destruction as well as inflammation of soft tissue. On T1-weighted sequences, SCC and metastatic lesions are hypointense [19,23].

### 1.6. Treatment Options in MU

No definitive treatment exists for SCC, but many treatment options can be used in combination resulting in healing rates up to 90% [27]. MUs, compared with other skin neoplasms, are more aggressive, thus treatment options must be well planned to benefit patient’s chances for survival. The most important prognostic factor is metastases: regional lymph nodes may be affected in 20%–66% and distant in 14% of cases [23,36]. Local treatment options, such as wide local excision and en bloc excision of lymph nodes, are the most frequent methods. Further options are electrocauterization of the base, cryologic surgery, intralesional injection of interferon alfa 2b or proximal amputation [27]. If recommended surgical margins (2 cm of normal-appearing tissue) [36,37] could not be retained in case of advanced lesion (e.g., bone or joint involvement), amputation of the extremity is the last curative option. Additional treatment options such as radio- and/or chemotherapy in terms of neoadjuvant or adjuvant therapy is recommended in patients with inauspicious prognostic factors or distant metastases [21,24,32,38]. Especially, pressure ulcers located at the sacral or iliac areas have extensive lymphatic drainage into the pelvis, which explains the frequent local and distant metastases. Lymphadenectomy is therefore an inevitable element of radical surgery if cancer progression is confirmed [38]. The remaining surgical wound should be covered with split-skin graft or soft tissue flaps if primary closure is not possible. This prevents recurrence of the malignancy [23].

## 2. Patient’s Characteristics

### 2.1. Case 1

**Figure 3:**
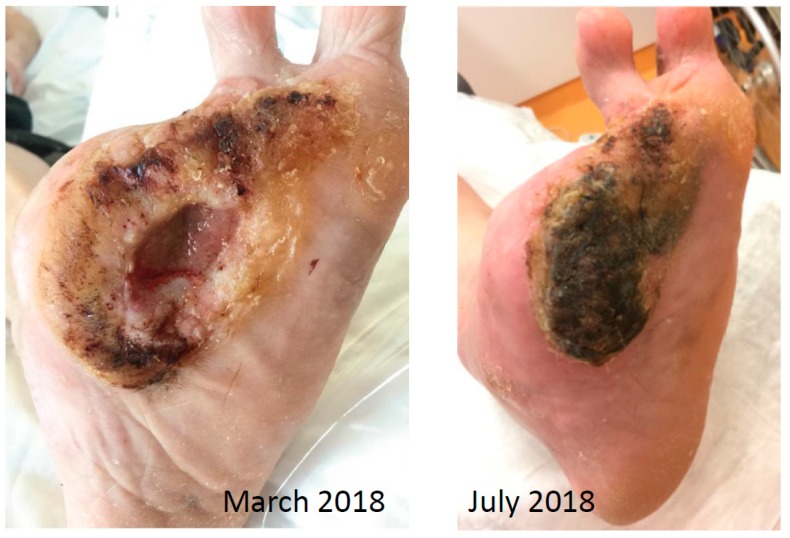
Clinical findings in patient 1.

The 67 year old male patient presented himself with a chronic ulcer on his left forefoot existing for about 8 years. He could not remember if it had ever healed completely, despite an adequate offloading in orthopedic shoes (Figure 3). In addition to his ulceration, he suffers from PAD, diabetes mellitus type 2 associated with retinopathy, nephropathy stage G4A2 and steatosis hepatis, coronary heart disease (CHD) and gout.

Because of fever and elevated inflammation markers at the time of admission, we initiated antibiotic treatment with ciprofloxacin. The microbiological testing revealed a mixed infection with *E. coli*, *Pseudomonas aeruginosa*, *Staph. aureus* and anaerobic species. Under the antibiotic treatment, inflammation markers decreased. Under effort of negative pressure wound therapy (NPWT), there was only little improvement in wound healing, so we decided to do histological testing of the ominous verrucous wound margin. The histological sample showed a highly differentiated SCC. In conventional X-rays, there were not any signs of bones or joint involvement. After successful abatement of the acute infection, he was planned for surgical resection.

### 2.2. Case 2

**Figure 4:**
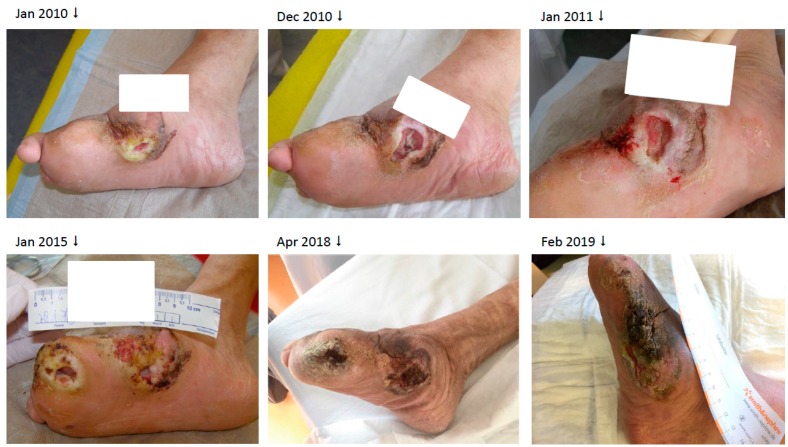
Chronic ulcer at the right feet and its variation during the years.

The 72 year old gaunt woman suffered from a chronic neuropathic ulcer of her right foot for now about 8 years. She was in our treatment repeatedly during this time (Figure 4). In addition to the DFU, her medical history showed progressed PAD, arterial hypertension and mild pronounced diabetes (A1c 5.9%) associated with neuro- and nephropathy stage G3bA2 and reactive depression. She had already undergone several amputations and revascularizations on both feet, was malnourished and admitted continuous smoking. For longer distances, she was wheelchair-bound. Figure 4 shows the development of the ulcer at the medial right foot during the years, since 2010.

**Figure 5:**
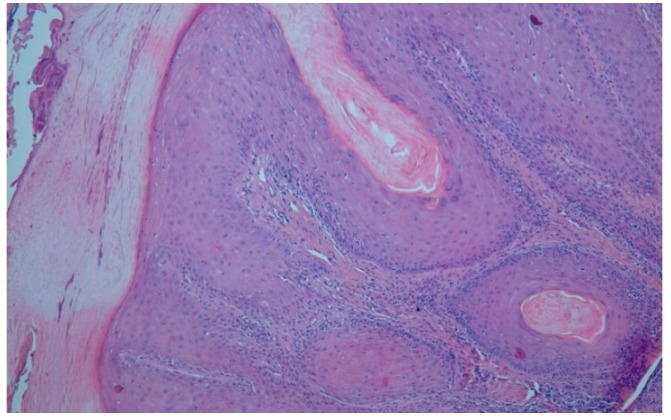
Histological imaging of the Ackermann carcinoma in Case 2. By courtesy and permission of Prof. Dr. P. von den Driesch, Department of Dermatology, Stuttgart General Hospital.

In May 2018 the chronic ulcer was sampled for the first time because of its suspicious verrucous appearance. In the histological samples, the subtype of a highly differentiated SCC, an Ackerman carcinoma, could be revealed (Figure 5). At that time, we avoided a total resection because the patient was contemporaneous in treatment with a complicated and long-standing gangrene on her left foot. After successful treatment of the gangrene, she was finally discharged to outpatient treatment and subsequent resection was planned when she would be recovered.

## 3. Clinical Findings and Treatment

### 3.1. Case 1

**Figure 6:**
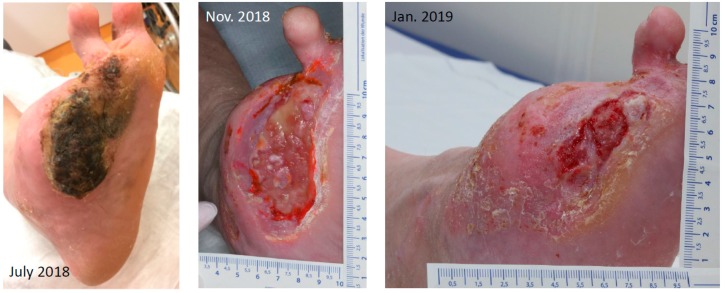
Pre- and postoperative clinical findings in Case 1.

**Figure 7:**
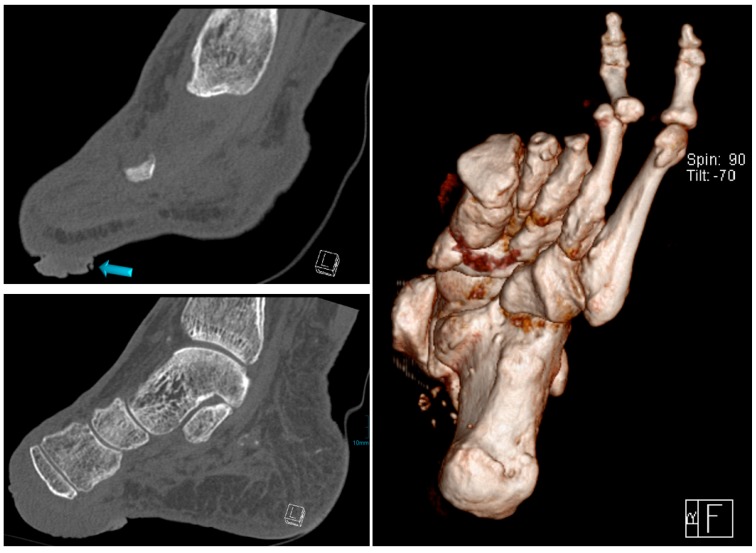
Radiological imaging of the foot in Case 1, arrow shows verrucous tumor mass.

In September 2018, the patient received deep excision of his carcinoma (Figure 6). The histopathological findings showed ulcerated, highly differentiated carcinoma spinocellulare (SCC) with a thickness of 7 mm, correspondent to a tumor grading G1. There was no evidence of lymph node metastases or bone involvement (Figure 7). A second resection was necessary in October 2018 because the excision borders were not reliable in sano. After the second surgical excision, there was no further proof of tumor relapse or progress. In November 2018, the excision wound could be covered with split-skin graft with good result. The final tumor formula was: pT3, cN0, cM0, R0, G1.

### 3.2. Case 2

**Figure 8:**
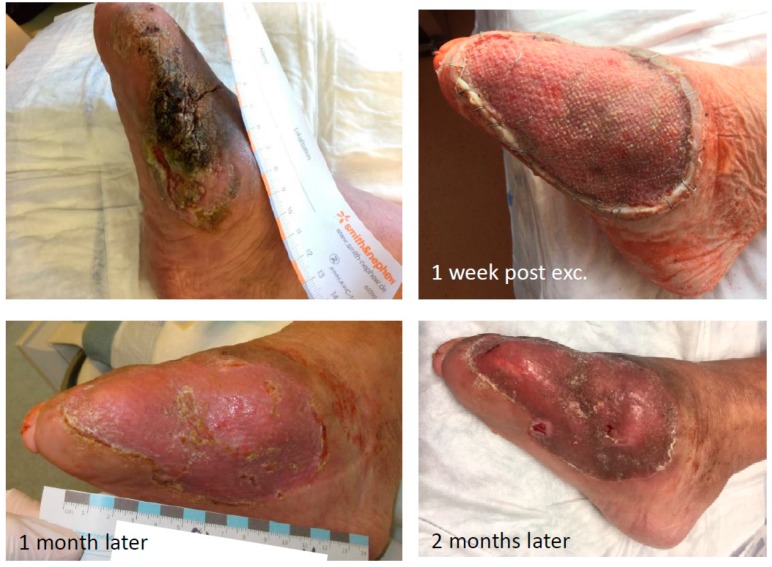
Pre- and post-op. findings.

**Figure 9:**
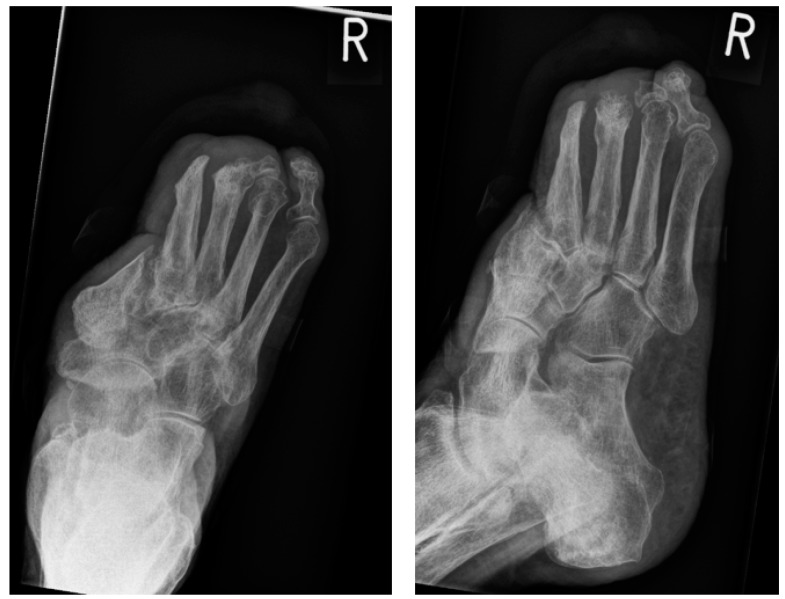
Radiological imaging.

In February 2019, she was admitted once more with recurring infections and foetid secretion in the area of the cancer. Thus, after an initial antibiotic treatment with piperacillin/tazobactam, because of detection of *Staph. aureus* and *Pseudomonas aeruginosa*, she was planned for subsequent tumor excision. After the primary excision, the hyper- and acanthotic SCC reached the resection margins. Thus, a second resection was necessary, which showed tumor free margins. The unconcealed excision wound was finally covered with a split-skin graft and additional negative pressure wound therapy directly post-operative (Figure 8). The sentinel lymph nodes were inconspicuous and the radiological plain film images did not show any involvement of the bone (Figure 9). We did not perform any further MR-imaging because the resolute lady categorically refused any further incisive surgery. Thus, finally the tumor formula was pT3, cN0, M0, R0, G1.

## 4. Outcome and Discussion

Marjolin’s ulcer (MU) is a rare but aggressive skin cancer and arise in chronic ulcers caused by vascular insufficiency, diabetic neuropathy, pressure or hemoglobinopathy or in scar tissue. A plethora of cytokines in the chronic wound maintain a chronic inflammation and can have stimulating effects on mitosis and migration of keratinocytes. MUs that develop from a pressure wound typically are more aggressive than those developing from other chronic wounds [34]. Patients with impaired immune system like diabetics have an increased risk of malignant transformation in their wounds [26]. However, the incidence of MU is greatest in the lower extremities (53.3% of MU) followed by the upper extremities (18.7% of MU) [29], but generally may occur at any anatomic location [19]. SCC is one of the most common skin cancer and Tobin et al. reported that 71% of MU can progress into SCC [28]. In our in-patient department, we annually treat about 360–400 patients with DFU and could detect two cases with malignant transformation, accounting for an annual incidence of 0.6%, or 6 per 1000 patients; however, in a specialized hospital unit with selected patient collective, it might be much lesser in outpatient treatment or general medicine.

If there are any clinical findings for malignant growth in a chronic wound, such as nodular or verrucous accretion, induration, contact bleeding, absent tendency of healing or even increase in surface, diagnosis should be confirmed by taking histological samples. It is necessary for all healthcare professionals of different specialties to face the possibility of malignant transformation in a chronic wound. Whenever the wound causes concern, a histological sample should be taken. Determined standardized biopsy procedures help to reduce false negative results. Therefore, Berkwits et al. and Lawrence recommend to take histological specimens from center and margin of a wound to reduce false negative results [39,40].

After confirming diagnosis metastases must be excluded, as they are crucial for prognosis and negatively impact survival; metastases to regional lymph nodes probably lead to death within 2–3 years [41]. Metastases at time of diagnosis are rather frequent and are found in 27% of patients [14,34]. The metastatic rate is increased in chronic wounds due to venous insufficiency and pressure wounds [26]. The rate of metastasis in SCC of the lower extremities was reported with about 30% [27]. A size >2 cm, rapid growth, ulceration, blurred borders, perivascular, intravascular, or deep subcutaneous infiltration are attributed with high-risk SCC [35]. Distant metastases typically affect brain, lung, liver, kidney and lymph nodes [26]. In our two cases, there was no hint of lymph node or distant metastases.

Pedal manifestation of SCC is rare. There are several additional reports about successful treatment of SCC of the lower extremity: In 2017, Raymond Cavalière reported an 83 year old female patient with successful wide excision of a SCC in an ulceration of her right heel [19]. In 2014, José Andrés Garcia-Marín reported two patients treated in a diabetic foot unit, a 71 and 56 year old man with actually no DFU [42]. First, the 71 year old man had an electrical accident during electrical storm when he was 40 years old. The lightning entered him occipital and leaved him through his right foot. In our opinion, the wound therefore must be classified as scar after electrical accident, no diabetes was reported. Secondly, the 56 year old man was immobilized because of paraplegia after a road accident 20 years ago and developed an ulcer of his right heel. This wound must be classified as pressure ulcer (PU), no diabetes was mentioned. In 2004, Christopher et al. had already reported the case of a 66 year old diabetic with a SCC in a scald burn scar of his right foot that did not heal for about 15 years. Finally, he underwent a below-knee amputation [43].

Thus, SCC in DFU remain seldom and we therefore report two cases with malignant transformation in DFU and their successful treatment. The diabetic foot is predisposed for malignant growth because of a chronic inflammatory status and repetitive tissue damage due to loss of protective sensation. Inflammation and repetitive mechanical tissue damage lead to chronic irritation and promote malignant growth. Additionally, malignant ulcers can appear with bacterial infection and fetid secretion, what may conceal malignant growth and delay clarification, as our cases and the case of Christopher et al. [43] show.

No definitive treatment for SCC has been established yet. A variety of treatment options is used and combined, inducing a healing rate up to 90% [27]. Standard procedures consist wide local excision, lymph node dissection and/or radiotherapy and chemotherapy. However, the wide excision with safety distance of 1–2 cm is the most commonly used treatment option [19] and enable preservation of extremity. Especially in elder people major amputation can lead to further immobility and make them often wheelchair-bound with negative effect on their quality of life and self-help competence. In our two cases, it was feasible to preserve extremity and attain R0-resection although a follow-up resection was necessary in both. Nonetheless, these results must be interpreted cautiously because we cannot present long-term results.

To prevent neoplasm recurrence, the surgical site should be covered by a graft or flap [26]. Sometimes excision may remain incomplete, as Mirshams reported a rate of 37.5% incomplete excisions in 2011 [44]. He defined the major risk factor of incomplete excision as tumor localization. Especially when located at lips, genitals, foot, forehead, cheek, nose and ear, extent of excision is restricted by specific anatomic conditions. Finally, amputation is the most definitive remedy, especially in the case of affected bones or joint involvement [45]. The two cases reported by José Andrés Garcia-Marín [42] both received infracondylar amputation; one because of bone involvement and lacking chance of revascularization and the other presumably because bedfast diseased without involvement of bones. The patient reported by Christopher et al. had a below-knee amputation as well [43]. In our cases, there was no evidence of deep infiltration, bone or locoregional lymph node involvement in conventional x-rays and ultrasound. As such, we performed extensive excision and split-thickness skin grafting (mesh graft) and could preserve the extremities with finally good outcome.

Ackerman carcinoma was first described by Lauren Vedder Ackerman in 1948. It is a sort of squamous cell carcinoma (SCC) with low malignancy and a characteristic verrucous surface, typically found after chronic irritation. The term ‘Epithelioma cuniculatum’ is originally described as a verrucous carcinoma of the sole and may also affect the thenar [46]. In the context of an ulcer of the shank, this carcinoma is called ‘Papillomatosis cutis carcinoides Gottron’. This entity was proven in Case 2.

Patients after SCC lesion possess a risk of 40% to develop further SCC lesions within the next 2 years. A tumor size >2 cm doubles the risk of recurrence and triples the metastatic rate [35]. Thus, a careful after-care of patients that have undergone surgery is mandatory. The 5 year survival of patients with SCC occurring in diabetic ulceration is about 55% (40%–69%) [19,26]. In patients that have undergone wide excision or proximal amputation the 5 year survival was denoted 60 and 69% respectively [26]. This, in our opinion, reflects that patients with DFU rather die of cardiovascular complications due to their diabetes than of SCC. Patients with DFU normally bear multiple chronicle illnesses, especially cardiovascular diseases, which let them die of macrovascular complications. Likewise, Raymond Cavalière’s reported female patient died of vascular complications 22 months after successful resection of her SCC [19]. Any further data of our patients are urgently necessary to estimate life expectancy and risk of recurrence.

## 5. Conclusions

SCC is the most common malignant tumor found in chronic wounds and shows infiltrative growth and tendency of metastasis. Its incidence of presenting in the lower extremities was reported ~1.5%.

The bio-environment in chronic wounds support malignant development by an imbalance of an abundance of cytokines, which enable on the one hand cell proliferation and migration and on the other maintain degeneration of extracellular matrix by an increase of MMPs. The physiology in wound healing and cancer progression resemble each other along general lines [5]. Therefore, chronic wounds prepare a breeding ground for malignant transformation, and a Marjolin’s ulcer sprouts. The general course from chronic wound or scar to the point of carcinoma was known long ago, but since 1850 it has been attributed to Jean N. Marjolin and called ‘Marjolin’s ulcer’.

We present two cases with long lasting DFU in a male and a woman, who developed malignant transformation in their chronic wound and were successfully treated with complete excision and split-thickness skin grafting (mesh graft) under preserving their extremities. There was no evidence of regional or distant metastases. Therefore, the rate of metastasis may be lower than in MU of other origin.

Physicians should always consider the possibility of malignant transformation in chronic wounds if there are several attributes such as absent healing or even increase of ulceration (>3 months) despite of optimal wound care, verrucous everted wound margin, excessive granulation tissue, secretion, contact bleeding or foul smelling exudate [26]. Pain, in our opinion, is not a reliable marker in DFU because of a nearly always existing painlessness due to diabetic polyneuropathy. There are a lot of causes for non-healing ulcers in diabetics like inadequate off-loading strategies, bacterial infection or PAD, that are often blamed for absent healing by health care professionals. Although development of SCC in DFU is rare, early biopsy should be aimed to confirm diagnosis whenever the wound gives cause for concern. A multidisciplinary team should assist in diagnosis, treatment and prevention of recurrence and metastases of SCC in DFU.

## Figures and Tables

**Table 1:** TNM-classification of SCC.

Criteria	Meaning
T1	Tumor extent ≤2 cm
T2	Tumor extent >2 cm and <5 cm
T3	Tumor extent ≥5 cm
T4	Tumor affects deeper structures such as bone, cartilage or muscle
N0	Not any lymph node metastases.
N1	Local lymph node metastases
M0	Not any metastases
M1	Distant metastases

## Abbreviations

CHD	coronary heart disease
DFU	diabetic foot ulcer
EGF	epithelial growth factor
FGF	fibroblast growth factor
HGF	hepatocyte growth factor
IL	interleukin
MMP	matrix metalloproteinase
MR-imagine/MRI	Magnet-resonance imaging, MRT
MU	Marjolin’s ulcer
PAD	peripheral arterial disease
PDGF	platelet derived growth factor
SCC	squamous cell cancer
TGF	transforming growth factor
TIMP	inhibitors of MMP
TNF	tumor necrosis factor
BCC	basal cell carcinoma
